# Bronchopulmonary foregut malformation with severe hemoptysis in advanced age: a case report and literature review

**DOI:** 10.3389/fmed.2024.1268008

**Published:** 2024-02-07

**Authors:** Cheng-Sen Cai, Bin Li, Yu-chun Yuan, Li-na Fu, Xiao-ye Zhang, Jun Wang

**Affiliations:** ^1^Department of Respiratory and Critical Care Medicine, Second Affiliated Hospital of Shandong University of Traditional Chinese Medicine, Jinan, Shandong, China; ^2^Department of Radiology, Second Affiliated Hospital of Shandong University of Traditional Chinese Medicine, Jinan, Shandong, China; ^3^Department of Pathology, Second Affiliated Hospital of Shandong University of Traditional Chinese Medicine, Jinan, Shandong, China

**Keywords:** BPFM, hemoptysis, advanced age, case report, literature review

## Abstract

Bronchopulmonary foregut malformation (BPFM) is a rare developmental malformation disease due to embryonic defects, with an even rarer occurrence in adults. We report a diagnosed case in an adult patient, and notably, this is the first reported case of such advanced age. Additionally, she experienced coughing up approximately 1 liter of blood and partial lung tissue, accompanied by respiratory failure and shock. Following treatment with transcatheter arterial embolization, her condition improved, and she has remained stable during follow-up. We present a case report and conducted a systematic review on this particular case.

## Introduction

Bronchopulmonary foregut malformation (BPFM) is a developmental malformation of the respiratory and digestive systems (1). It is caused by budding defects, differentiation, and abnormal separation of the embryonic foregut, ultimately manifesting in isolation of the lung and bronchial–esophageal fistula formation. As a rare disease, it is primarily diagnosed in childhood. We report the case of one elderly patient with BPFM. She presented with significant hemoptysis and expectorated a clot that was confirmed to contain lung tissue. She later developed respiratory failure and hypovolemic shock. A chest CT showed anomalous masses in the lower lobe of the right lung, aortography detected an abnormal feeding artery, and esophageal angiography revealed an esophageal–bronchial fistula. The patient’s condition was stabilized after artery embolism and comprehensive treatment.

## Case report

The patient, a 67-year-old woman, was admitted to the hospital for 5 days with hemoptysis and a history of repeated cough over many years. She had a history of drinking approximately 100 g per day for 15 years, with no other remarkable personal or family history. Moist rales were found in the lower lobe of the right lung.

**Figure 1:**
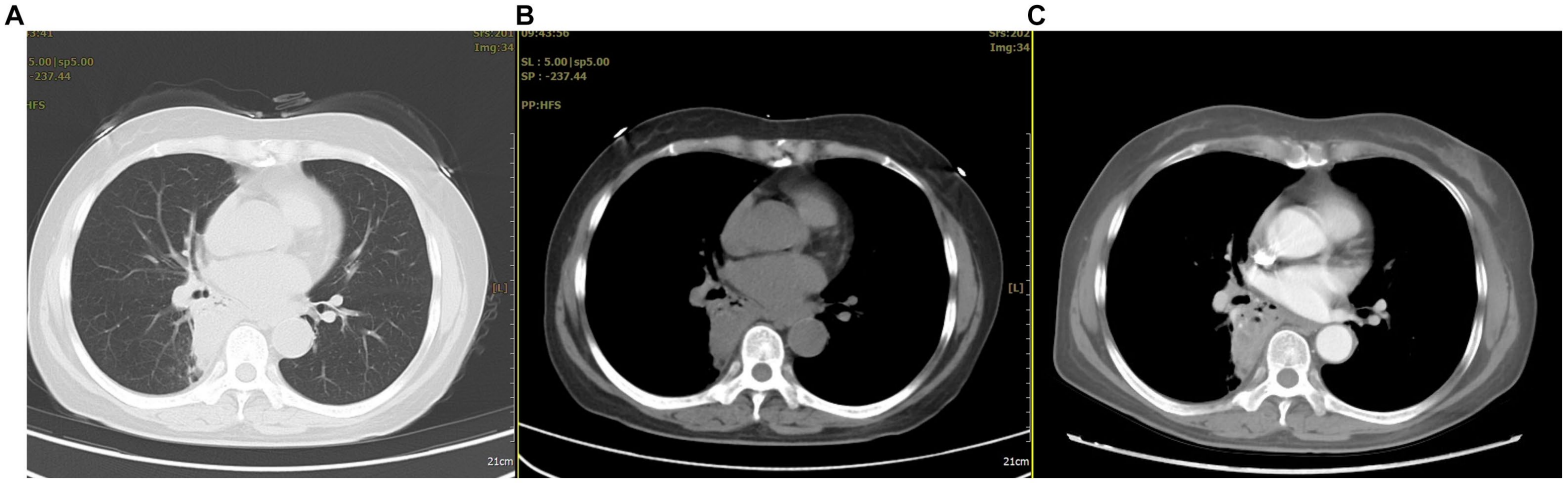
The chest CT revealed abnormal lesions in the lower lobe of the right lung. **(A)** In the lung window, a consolidation shadow was observed in the posterior segment of the right lower lobe. **(B)** In the mediastinal window, the lesion appeared to have regular margins. **(C)** After contrast enhancement, heterogeneous enhancement was noted within the lesion.

After admission, multiple laboratory tests were arranged. The patient’s white blood cell and neutrophil counts were 11.1*10^9^/L and 7.1*10^9^/L, respectively; her D-dimer level was 1.97 mg/L; her procalcitonin level was normal; and her tuberculin test and tumor-related indicators were negative. A chest CT showed a nodular shadow with a relatively regular morphology and uneven enhancement in the right lung (Figure 1).

Fiberoptic bronchoscopy showed obvious local congestion of the mucosa in the right lower lobe, and the lumen of the posterior segment of the right lower lobe was suspected to be blocked by a neoplasm. A brush biopsy was taken for pathological examination which later indicated inflammation; approximately 100 mL of bleeding occurred during the process, which was treated with local hemostasis.

Upon differential diagnosis, hemoptysis-causing diseases such as lung malignancy, tuberculosis, and pulmonary embolism were ruled out. A normal cranial CT excluded cough caused by swallowing reflex dysfunction. Thus, evaluation of the patient only revealed mild pulmonary inflammation.

**Figure 2:**
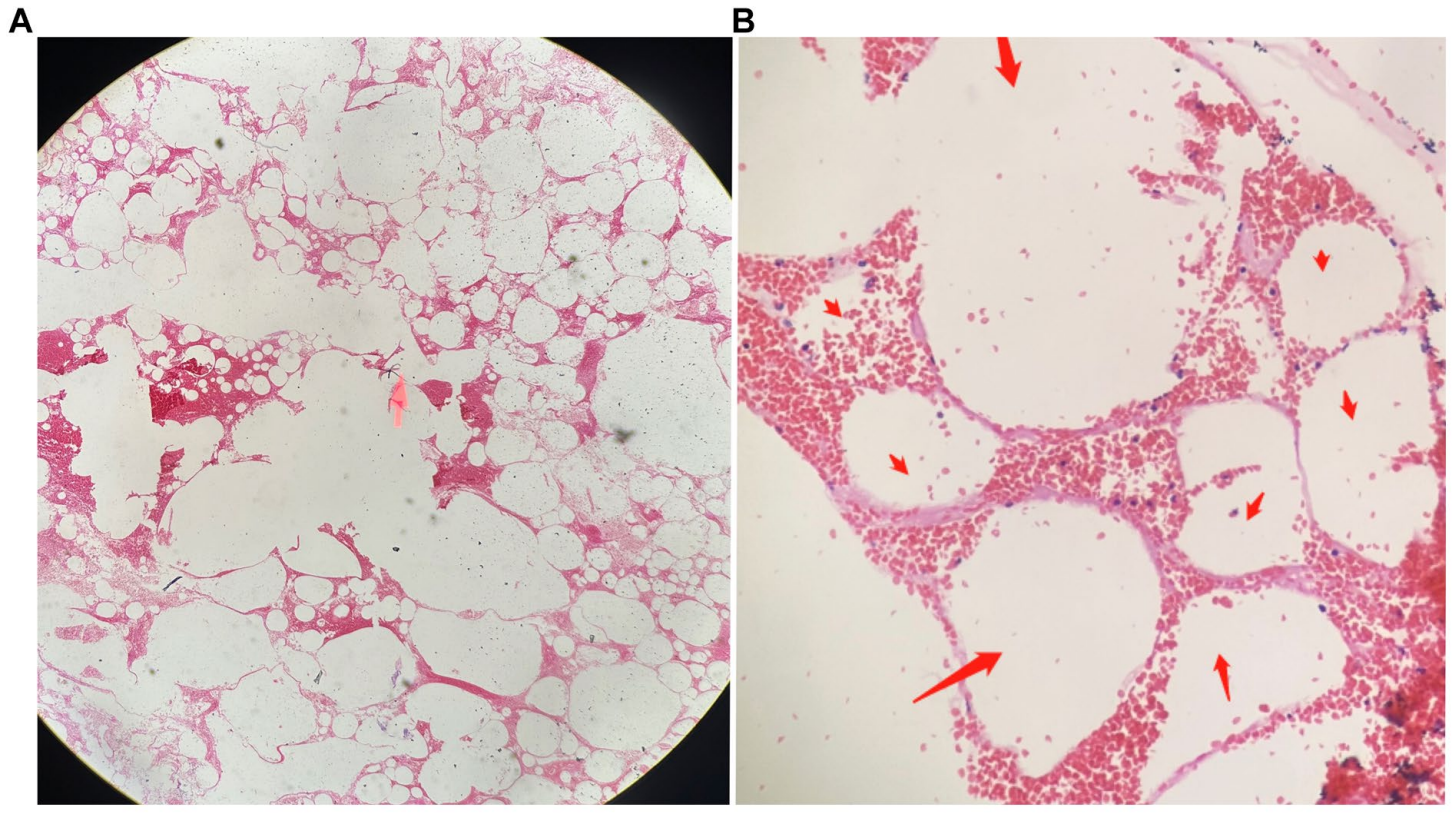
The pathological findings of the expectorated mass on HE staining. **(A)** Dilated and degenerated alveolar contours are visible in the field of view (HE, ×40). **(B)** Interstitial congestion and loss of alveolar epithelial cells can be observed (indicated by red arrows; HE, ×100).

During treatment, the patient had two episodes of massive hemoptysis. During the first episode, the patient expectorated approximately 300 mL of material, including clots, which was later sent for pathological examination. Hematoxylin and eosin (H&E) staining showed degenerated alveolar tissue structure (Figure 2).

**Figure 3:**
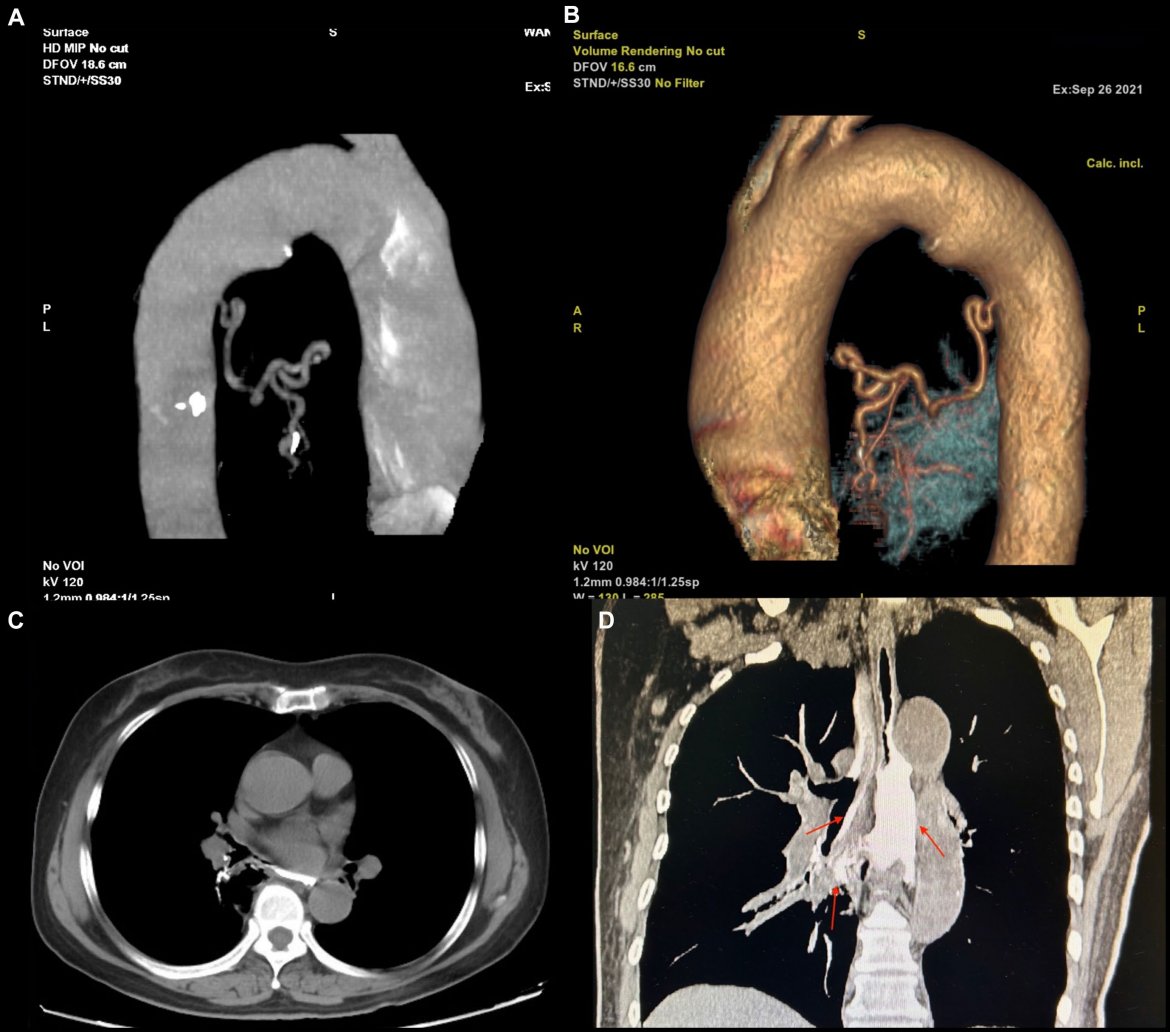
**(A,B)** The blood supply vascular reconstruction of the lesion reveals a large, tortuous, and malformed artery originating from the descending aorta, extending toward the site of the lesion. **(C,D)** The upper gastrointestinal (UGI) contrast study. **(A)** In the axial view, an abnormal communication between the trachea and esophagus is observed. **(B)** In the coronal view, the contrast agent is seen entering the bronchus through the fistula and remaining within the airway (indicated by a red arrow).

Although the bleeding stopped temporarily after hemostasis treatment, the patient had another episode of massive hemoptysis around 8 h later, expectorating a volume of approximately 700 mL. She subsequently developed confusion, respiratory failure, and hemorrhagic shock. We immediately arranged for a blood transfusion and mechanical ventilation and performed an emergency vascular intervention. Body arterial angiography revealed a twisted and deformed artery originating from the thoracic aorta that was supplying blood to the lesion in the right lower lobe of the lung (Figures 3A,B).

Therefore, arterial embolization of this blood vessel was performed. After polyvinyl alcohol particle embolization (particle size: 350–560 μm), the hemoptysis stopped abruptly. The patient recovered soon after basic treatment and the ventilator was removed. In addition, upper gastrointestinal (UGI) radiography revealed an abnormal connection between the bronchus and esophagus (Figures 3C,D), so we diagnosed the patient with BPFM.

**Figure 4:**
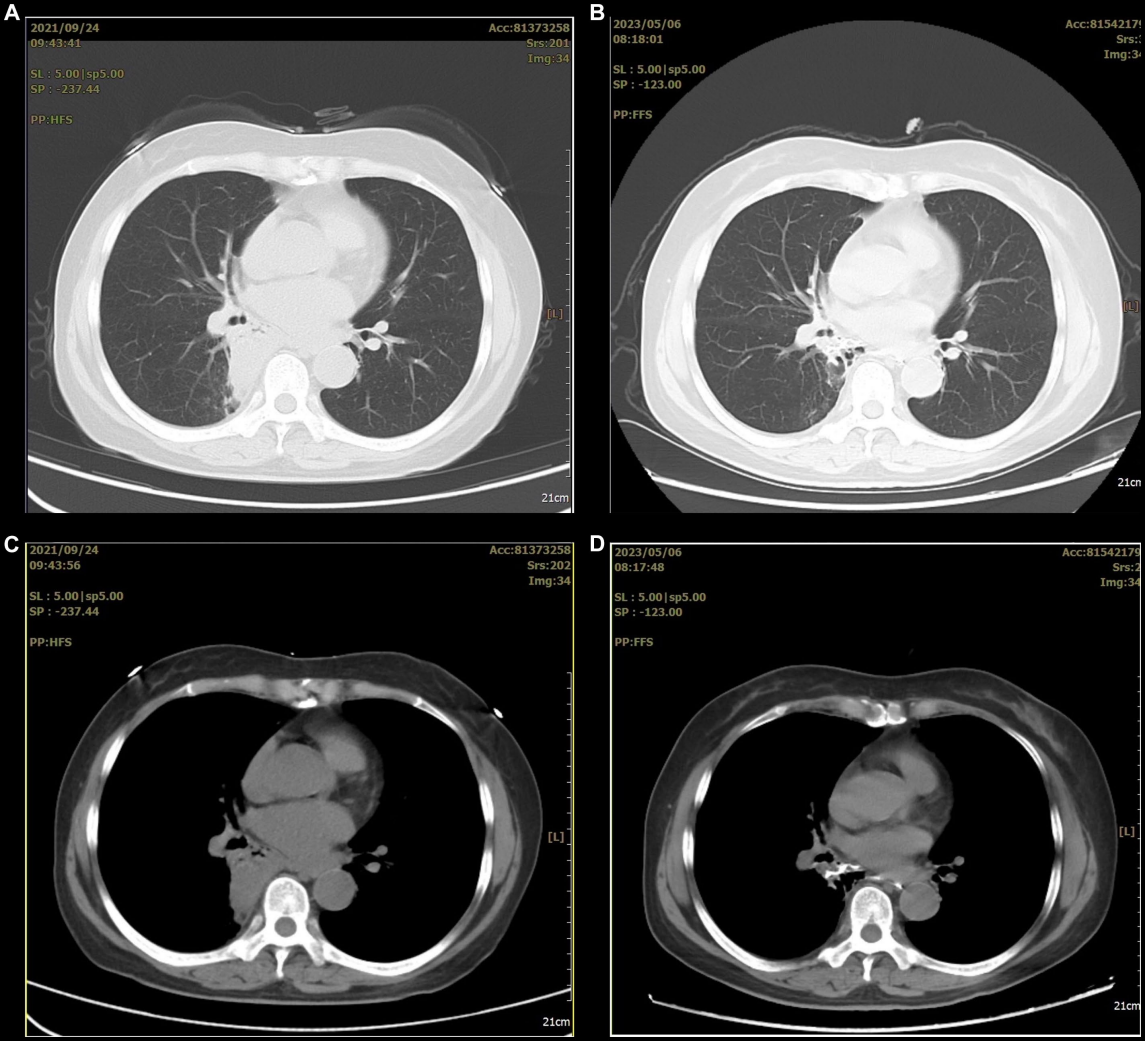
The before and after comparison of the arterial embolization treatment for the lesion. **(A,C)** A consolidation shadow can be seen in the right lung before the treatment. **(B,D)** After the treatment, the consolidation shadow appears significantly reduced.

However, she refused surgical treatment due to fear of the risks associated with her advanced age and chose to be discharged. Therefore, we advised her to avoid a stimulating diet, especially alcohol. Assessed through telephone follow-up, the patient’s condition remained stable after discharge, but occasional coughing still occurred. Eighteen months later, a chest CT showed that the lesion had significantly decreased in size and showed local cystic changes (Figure 4). Therefore, we remain concerned that the patient’s condition may worsen in the future.

## Discussion

BPFM is a subcategory of pulmonary isolation that was first described in 1968 (2). It occurs due to developmental abnormalities present during the fourth week of embryonic development. Srikanth et al. divide BPFM into four different groups. In group I (16%), the anomaly is associated with esophageal atresia and tracheoesophageal fistula. In group II (33%), one lung originates from the lower esophagus. In group III (46%), an isolated anatomic lung lobe or segment communicates with the esophagus or stomach. In group IV (5%), a portion of the normal bronchial system communicates with the esophagus (3).

Epidemiologically, patients often present with respiratory symptoms because of the abnormal entrance between the esophagus and bronchi. Therefore, most cases are diagnosed in childhood. However, a small proportion of patients have no obvious symptoms early in life and the condition is not detected until adulthood. We found fewer than 20 cases of adult BPFM in the literature. Two hypotheses may explain the absence of obvious symptoms in adults. The first is that the membranous tissue covering the fistula is perforated in adulthood, and the second is that the esophageal wall fold that overlaps the fistula loses mobility with aging (4). In the present case, the patient was 67 years old at the time of diagnosis, 3 years older than the oldest patient reported in the literature, thus providing new insight into the oldest possible age of presentation (5).

The symptoms of BPFM are atypical and vary according to age and disease subgroup (1). Generally, recurrent pneumonia or bucking when intaking fluid or food are common in childhood, whereas chest pain, dyspnea, and hemoptysis are common in adults (5–8). The pathological factors underlying the development of hemoptysis are not clear. We speculate that the abnormal entrance allows food particles to enter the bronchi, causing chronic infection and inflammation. Thus, some deformed blood vessels might be injured by physical and chemical stimulation of the lung, ultimately resulting in bleeding. In the present case, hemoptysis was the patient’s primary symptom; approximately 1 liter of blood had accumulated, causing respiratory failure and hypovolemic shock, which is rare. In particular, this patient expectorated a mass during one of her hemoptysis episodes. Necrotic alveolar structures revealed by H&E staining confirmed another diagnosis: necrotizing pneumonia. We considered that this chronic infection caused local lung tissue necrosis, as well as rupture and bleeding of the deformed, fragile blood vessel, all of which was ultimately expectorated like the old Chinese saying, “fruits fall off when ripe.”

As a congenital anomaly, BPFM can involve cystic lesions in the bronchial–esophageal junction, covered with squamous and columnar epithelium. This phenomenon demonstrates a close correlation between BPFM and abnormal embryonic development (9). Through operative pathology, Xie et al. found underdeveloped, structurally disordered lung tissue containing dilated cysts of varying sizes, necrosis, and abundant foam cells in the lumen (10). In the present case, we could only detect structurally disordered and degraded alveolar structures through pathological staining of the expectorated clots. Unfortunately, we did not obtain surgical pathology results because the patient refused surgery. However, this refusal did not affect the diagnosis of the disease.

The diagnosis of BPFM primarily relies upon imaging. Yang et al. reviewed 61 cases of BPFM and found that the diagnostic methods used were mainly UGI radiography (62.3%) and CT (11.5%) (7). Caro-Domínguez et al. reported that BPFM can also be diagnosed by ultrasound and MRI (11). A UGI series can detect abnormal bronchial–esophageal channels. According to Yutaka et al., CT scans reveal that approximately 75% of BPFM patients have lesions in the right lung, particularly in the posterior basal segment of the right lower lobe, and the typical manifestations are consolidation and cystic shadows (4). Additionally, systemic circulation arteriography can reveal abnormal artery blood supply at the focus, some of which is returned by the pulmonary vein (5, 8). In the present case, CT revealed a solid lesion in the lower lobe of the right lung, with a visible gas–liquid appearance. After absorption, the lesion formed a cystic shadow. UGI radiography revealed an esophageal–bronchial fistula and contrast agent could be seen flowing out of the bronchus. This patient presented with almost all of these symptoms.

With respect to treatment, because BPFM is primarily discovered in childhood, early surgery is the first-line treatment, after which the prognosis is good. However, given the possibility of significant surgical damage, some authors recommend artery embolization in later stages; however, this method cannot achieve a radical cure (12). Indeed, the outcomes of conservative management are poor and may result in repeated lung infection or even hemoptysis. If not properly managed, the patient’s life may be in danger. The patient in the present case was older at the time of onset and presented with massive hemoptysis, hypovolemic shock, and respiratory failure; approximately 800 mL of red blood cells were transfused accumulatively. The patient feared the risks of surgery, so she was treated by embolization. Although the treatment was effective and the lesion was relatively stable after reexamination, the patient’s cough persisted, indicating she may be at risk for a secondary infection.

To conclude, BPFM is a rare disease that primarily occurs in children. Bronchial–esophageal fistulae can be found through UGI radiography, chest CT shows lesions often located at the periphery of the right lung, and abnormal blood supply in the systemic circulatory arteries can be found by systemic circulation arteriography. This case and literature review provide us with indications that symptoms of BPFM become more severe in older patients, even to the point of life-threatening conditions. Early detection of BPFM can be achieved through chest CT or UGI radiography. Additionally, surgical intervention is the preferred treatment method for BPFM. In cases where surgery is not feasible, transcatheter arterial embolization can be considered as an alternative. However, it is important to note that this method does not provide a definitive cure for BPFM.

## Data availability statement

The original contributions presented in the study are included in the article/supplementary material, further inquiries can be directed to the corresponding author.

## Ethics statement

Written informed consent was obtained from the individual(s), and minor(s) legal guardian/next of kin, for the publication of any potentially identifiable images or data included in this article.

## Author contributions

C-SC: Writing – original draft, Writing – review & editing. BL: Data curation, Resources, Visualization, Writing – review & editing. Y-cY: Data curation, Visualization, Writing – review & editing. L-nF: Writing – original draft, Writing – review & editing. X-yZ: Data curation, Visualization, Writing – review & editing. JW: Funding acquisition, Writing – original draft, Writing – review & editing.
